# Silver nanoparticles nucleated in NaOH-treated halloysite: a potential antimicrobial material

**DOI:** 10.3762/bjnano.12.63

**Published:** 2021-08-05

**Authors:** Yuri B Matos, Rodrigo S Romanus, Mattheus Torquato, Edgar H de Souza, Rodrigo L Villanova, Marlene Soares, Emilson R Viana

**Affiliations:** 1 Departamento Acadêmico de Física (DAFIS-CT), Universidade Tecnológica Federal do Paraná - UTFPR - Curitiba, Brazil; 2 Departamento Acadêmico de Engenharia Mecânica DAMEC, Universidade Tecnológica Federal do Paraná - UTFPR - Curitiba, Brazil; 3 Secção de Engenharia e Ciência dos Materiais, Instituto Militar de Engenharia - IME - Rio de Janeiro, Brazil; 4 Departamento Acadêmico de Química e Biologia (DAQBI-CT), Universidade Tecnológica Federal do Paraná - UTFPR - Curitiba, Brazil

**Keywords:** antimicrobial activity, DIO coating, halloysite, nanocomposites, silver nanoparticles

## Abstract

Despite all recent advances in medical treatments, infectious diseases remain dangerous. This has led to intensive scientific research on materials with antimicrobial properties. Silver nanoparticles (Ag-NPs) are a well-established solution in this area. The present work studied the nucleation of silver on halloysite substrates modified by chemical treatment with NaOH. The resulting stabilized Ag-NPs were characterized by X-ray diffraction, transmission electron microscopy, and energy-dispersive X-ray spectroscopy. The nucleation was characterized by thermogravimetric analysis and differential scanning calorimetry. The antimicrobial properties of the Ag-NPs were investigated against *E. coli* and *S. aureus*. The potential of the Ag-NPs for industrial application was tested by dispersing them into low-density polyethylene. The importance of the chemical affinity between matrix and additive was tested through coating the Ag-NPs with dodecanethiol, a non-polar surfactant. The resulting composites were characterized by scanning electron microscopy and in terms of surface antimicrobial activity. The results demonstrate that the Ag-NPs synthesized in this work are indeed antimicrobial, and that it is possible to imbue a polymeric matrix with the antimicrobial properties of Ag-NPs.

## Introduction

The number of people dying from bacterial infections has been significantly reduced with the emergence of antibiotics, but some bacterial diseases are still amongst the most dangerous to human life. In addition, as antibiotics become more popular, bacteria are evolving to become resistant, a phenomenon known as “antimicrobial resistance” and listed by the World Health Organization as one of the top ten threats to public health [1]. Antimicrobial nanomaterials are one of the most promising antibiotic-free alternatives for many applications. Among them are metallic nanoparticles, which could be potent inorganic antimicrobial agents through ion release and the capability to rupture the cellular membrane and to disrupt internal cellular components, such as DNA [2–6].

Silver nanoparticles (Ag-NPs), in particular, are known for their antimicrobial properties and are one of the most extensively studied inorganic antimicrobial agents [7–9]. Early studies suggested that Ag-NPs strongly inhibit the growth of common microorganisms and that they may be used as an alternative way to overcome bacterial resistance to antibiotics [10–11]. This is due to a combination of antimicrobial mechanisms including the generation of reactive oxygen species (ROS) and the diffusion of silver ions (Ag^+^) into bacterial cells [12]. However, a large-scale industrial usage of Ag-NPs is still difficult because most synthesis techniques used in labs are unsuitable for commercial use. The main issues are high procedural complexity and elevated cost, which jeopardize scalability efforts fundamental to the industrial mass-production of Ag-NPs.

Amongst the most scalable synthesis routes for Ag-NPs, some techniques stand out by using stabilizers, such as PVP [13] or PEG [14] capping agents, to ensure that silver nucleates and stabilizes into nanosized particles, instead of dissolving back into Ag^+^ ions or growing up to the micrometer scale. Some routes stabilize Ag-NPs by nucleating silver into clay substrates [15–16], such as kaolinite [17–19], montmorillonite [18–21], and halloysite nanotubes [22–24]. The advantages are, for instance, preventing particle agglomeration, improving dispersability into polymeric matrices, keeping good biocompatibility with the human body, and immobilizing Ag-NPs on a substrate, which provides better interaction with bacteria and is a more eco-friendly way to obtain antimicrobial agents.

**Figure 1:**
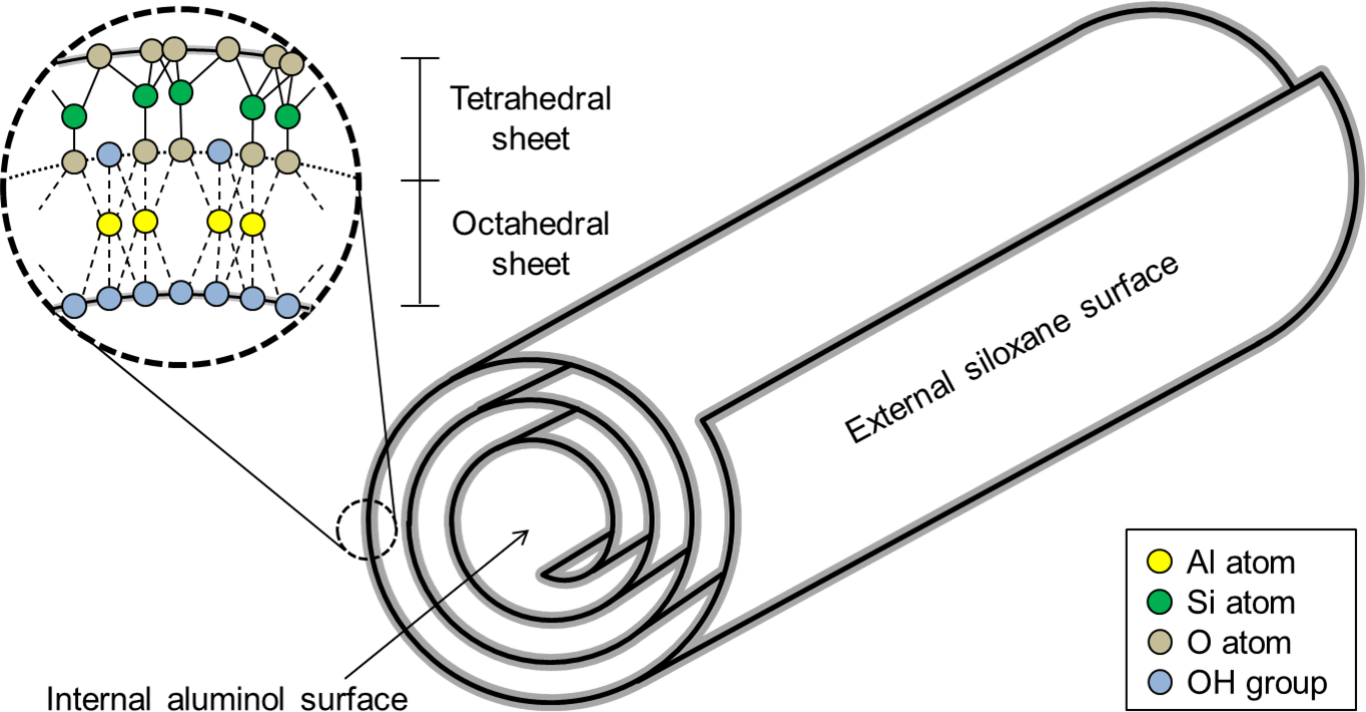
Crystal structure of halloysite particles.

Halloysite (HNT) is a natural clay, consisting of an aluminosilicate sheet that folds over itself in virtue of the internal stress inherent to the crystalline structure of the material, forming nanotubes [25]. As shown in Figure 1, it folds with a silicate phase facing outwards (Si-O), and an aluminol phase facing inwards (Al-OH). Since the internal and external surfaces of HNT have different chemical constitutions, they display different chemical affinities as well. Silver nucleation, for example, is favoured on the aluminol surface, due the high affinity between Ag^+^ ions and hydroxy groups (OH^−^) [26]. There is evidence suggesting that the silver nucleation on the external surface of HNT tends to occur in crystalline defects, where aluminol is exposed [23]. Synthesizing Ag-NPs supported by HNT has the advantage of improving stability, thus enhancing the antimicrobial properties of the material [22–24,27–28].

In this work we treated the HNT surface with NaOH to improve Ag-NP nucleation into the clay substrate. The resulting nanocomposite was evaluated in terms of chemical composition, morphology, and antimicrobial properties, while the nucleation process was characterized in terms of thermal behaviour and structural changes. We also investigated the potential of the Ag-NPs for industrial application by dispersing them into low-density polyethylene (LDPE) and evaluating the chemical compatibility between matrix and additive by testing a dodecanethiol (DIO) coating to improve the dispersion of the Ag-NPs into LDPE. The resulting composites were then evaluated in terms of surface antimicrobial activity.

## Experimental

Halloysite (*>*99%), silver nitrate (AgNO_3_(s), *>*99%), and dodecanethiol were obtained from Sigma-Aldrich; sodium hydroxide (NaOH(s), *>*99%) was purchased from Alpha Quimica; low-density polyethylene (LDPE) was purchased from Braskem.

Transmission electron microscopy (TEM) was performed in a Jeol JEM-1400 plus (480 keV), energy-dispersive X-ray spectroscopy (EDS) and scanning electron microscopy (SEM) in a Zeiss EVO MA15, and thermogravimetric analysis (TGA) and differential scanning calorimetry (DSC) in a simultaneous thermal analyzer Netzsch STA 449 F3. X-ray diffraction (XRD) measurements were carried out using a Shimadzu diffractometer, model XRD-7000, with Cu Kα radiation (λ = 0.154 nm).

### Substrate preparation

To prepare the substrates for silver nucleation, halloysite was treated in a NaOH(aq) bath. Three HNT suspensions were prepared sonicating 100 g of HNT powder in 400 mL of deionized water (DI) for 20 min at 20 kHz. One suspension, labelled HNT-0, was not treated with NaOH(aq) and was used as a control sample. To the other two suspensions, 200 g of NaOH were added and stirred until complete dissolution, followed by resting periods of four and eight days, resulting in the samples HNT-4 and HNT-8, respectively. After the resting periods, the three suspensions were filtered and extensively washed with deionized water to remove residual NaOH and then dried naturally. To characterize the prepared substrates, samples HNT-0, HNT-4, and HNT-8 were recorded with TEM, while sample HNT-8 was also measured with XRD. (XRD was also performed for HNT-0 and HNT-4 but omitted from this report because it did not show any significant difference from HNT-8).

### Silver nanoparticle synthesis

To synthesize silver nanoparticles, 10 g of HNT (from the three samples previously prepared: HNT-0, HNT-4, and HNT-8) were dispersed in 20 mL of AgNO_3_ solution (3 M) and left to rest for one day to load the silver nitrate into the clay structure. Then the samples were filtered, dried at 80 °C for 3 h, and heated to 500 °C for 15 min, to reduce AgNO_3_ into metallic silver. They were then labelled Ag/HNT-0, Ag/HNT-4, and Ag/HNT-8 (according to the NaOH treatment), and characterized by TEM and EDS. Sample Ag/HNT-8 was also characterized by XRD.

### Ag-NPs nucleation analysis

In order to study the nucleation of silver nanoparticles, 1 g of Ag/HNT-8 was used for TGA and DSC analysis, from room temperature up to 600 °C, at constant heating of 20 °C/min. In order to observe the phases identified in the DSC/TGA measurements, four 10 g samples of HNT-8 were loaded with AgNO_3_ using the process described in section “Silver nanoparticle synthesis” and heated up to 65, 105, 230, and 505 °C. After that, TEM images were obtained to correlate morphological changes occurring during the synthesis with the thermodynamic data from DSC/TGA analysis. Finally, another 10 g of HNT-8 was loaded with AgNO_3_ and heated up to 700 °C, with visual inspections being performed at 40, 100, 120, 200, 350, 500, and 700 °C, to evaluate the colour changes during heating.

### Minimal inhibitory concentration test

The bactericidal effect of Ag/HNT-8 and Ag/HNT-0 was evaluated by a minimal inhibitory concentration (MIC) test. The first step was to obtain pure strains of *Escherichia coli* (ATCC 25922) and *Staphylococcus aureus* (ATCC 29213). This was achieved by inoculating the bacteria in selective culture media (rapid coliform broth agar for *E. coli* and salted manitol agar for *S. aureus*), followed by 24 h of incubation at 37 °C. After incubation, five colonies of each bacterial species were inoculated into a general culture medium (Mueller–Hinton + agar broth) and incubated again at 37 °C for another 24 h. After the bacteria colonized their cultivation media, five isolated colonies of each species were selected and transferred to two tubes (one for each species) containing 9 mL of saline solution (8.5 mg/mL of NaCl). The two suspensions were then homogenized in a vortex mixer, and subsequently had their concentration adjusted to 10^8^ colony forming units (CFU) per millilitre, as recommended by [29].

The two bacterial suspensions were then further diluted, with liquid Mueller–Hinton broth, to a concentration of 10^5^ CFU/mL. These new 10^5^ CFU/mL suspensions were then divided into ten tubes each, and to each were added the following concentrations of Ag/HNT-0 or Ag/HNT-8: 3, 6, 12, 25, 50, 100, 200, 400, 800, and 1600 ppm. Finally, each nanoparticle + bacteria suspension was incubated again at 37 °C for 24 h. The minimal inhibitory concentration was determined examining bacterial growth at each nanoparticle concentration.

### Dispersion of Ag/HNT-8 in a LDPE polymer matrix

Ag/HNT-8 was also tested as an antimicrobial additive to LDPE and the biocide properties of the resulting composite were quantified by means of an antimicrobial surface activity test. First, two 10 g samples of Ag-NPs were prepared according the process described in section “Silver nanoparticle synthesis”. Then, one of the Ag-NP samples was coated with DIO to make its surface hydrophobic and therefore more chemically compatible with LDPE, a non-polar polymer matrix. This coating was performed suspending the to-be-coated sample in 10 mL of ethanol/dodecanethiol mixture (200:1, v/v) for 24 h, followed by filtering, washing with ethanol, and drying at 60 °C for 2 h. The coated sample was labelled Ag/HNT-8/DIO, and the DIO coating was considered successful since qualitative tests showed that Ag/HNT-8/DIO did indeed present hydrophobic behaviour, while Ag/HNT-8 did not.

Afterwards, 2 g of Ag/HNT-8 and Ag/HNT-8/DIO were mixed with 650 g of LDPE pellets. Both mixtures were homogenized and moulded into square plates of 8 × 8 cm^2^ (95 MPa, 860 mm/s, 1 m double screw, 170 °C injection temperature, 90 °C mold temperature). Polymer plates doped with Ag/HNT-8 and Ag/HNT-8/DIO were characterized by SEM. LDPE plates without any additive were also prepared to be used as control in the antimicrobial test.

**Figure 2:**
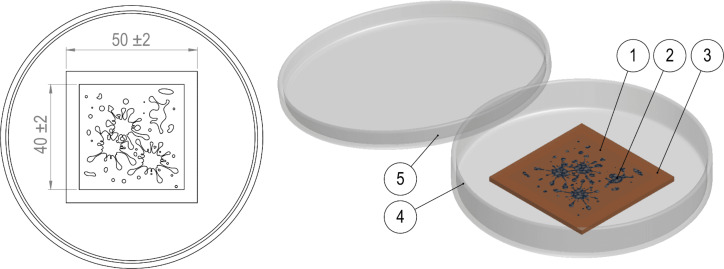
Layout of sample dimensions (in mm × mm) and preparation for the polymer antimicrobial test showing (1) film cover, (2) bacterial suspension, (3) LDPE test specimen, (4) Petri dish, and (5) Petri dish cover.

Finally, antimicrobial surface activity tests were performed for LDPE samples doped with Ag/HNT-8 and Ag/HNT-8/DIO. The tests were performed following the guidelines specified in the JIS Z2801 standard, and consist of preparing *E. coli* and *S. aureus* suspensions (analogous to the ones produced in the MIC analysis) and exposing them for 24 h to the surface of the LDPE plate. Figure 2 shows the experimental layout described above. After incubation, the suspensions were carefully washed away and re-inoculated in the agar cultivation media. The inhibitory activity of the plastic surface was quantified by comparing the number of viable colonies from the suspensions that were exposed to treated LDPE with the number of viable colonies from the suspensions that were exposed to the control polymer.

## Results and Discussion

### HNT substrates and Ag-NPs characterization

**Figure 3:**
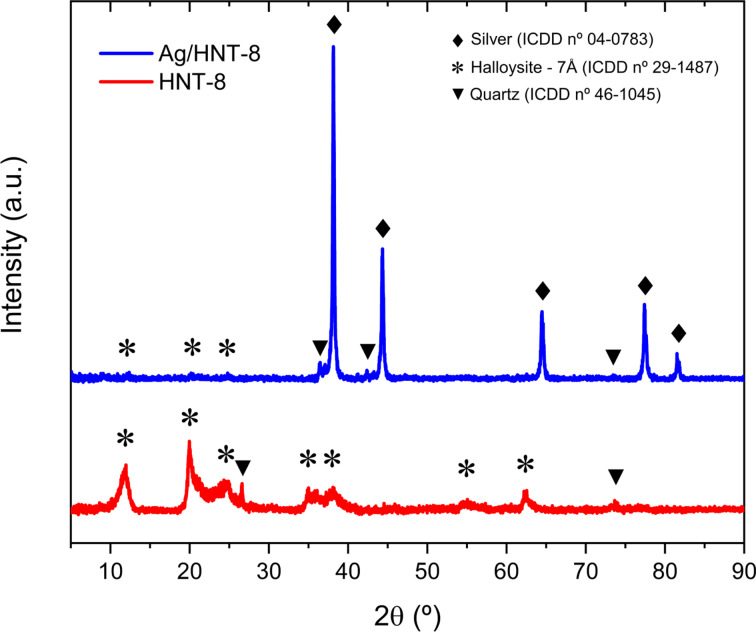
XRD patterns of HNT-8 and Ag/HNT-8.

Figure 3 shows the XRD patterns of HNT-8 and Ag/HNT-8. Diffraction peaks of HNT-8 were found at 2θ = 11.66°, 20.09°, 23.88°, 35.09°, 37.81°, 54.82°, and 62.47°, matching the (001), (100), (002), (110), (003), (210), and (300) planes of 7Å dehydrated halloysite (ICDD 29-1487). Additional peaks were found at 2 θ = 26.57°and 76.80°, matching the diffraction pattern of quartz (ICDD 46-1045), a commonly found secondary phase in HNT samples [30]. This result confirms that the substrate is indeed halloysite clay and the absence of any NaOH phase indicates that the treatment waste was successfully washed away. For Ag/HNT-8, diffraction peaks were found at 2θ = 38.12°, 44.29°, 64.44°, 77.39°, and 81.53°matching the (111), (200), (220), (311) and (222) planes of FCC metallic silver (ICDD 04-0783) with a lattice constant of 4.089 Å. Only three halloysite peaks were found in Ag/HNT-8, corresponding to the (001), (100), and (002) planes, and their intensity was notably smaller than that of the silver peaks, suggesting no stacking of halloysite layers in the composite.

**Figure 4:**
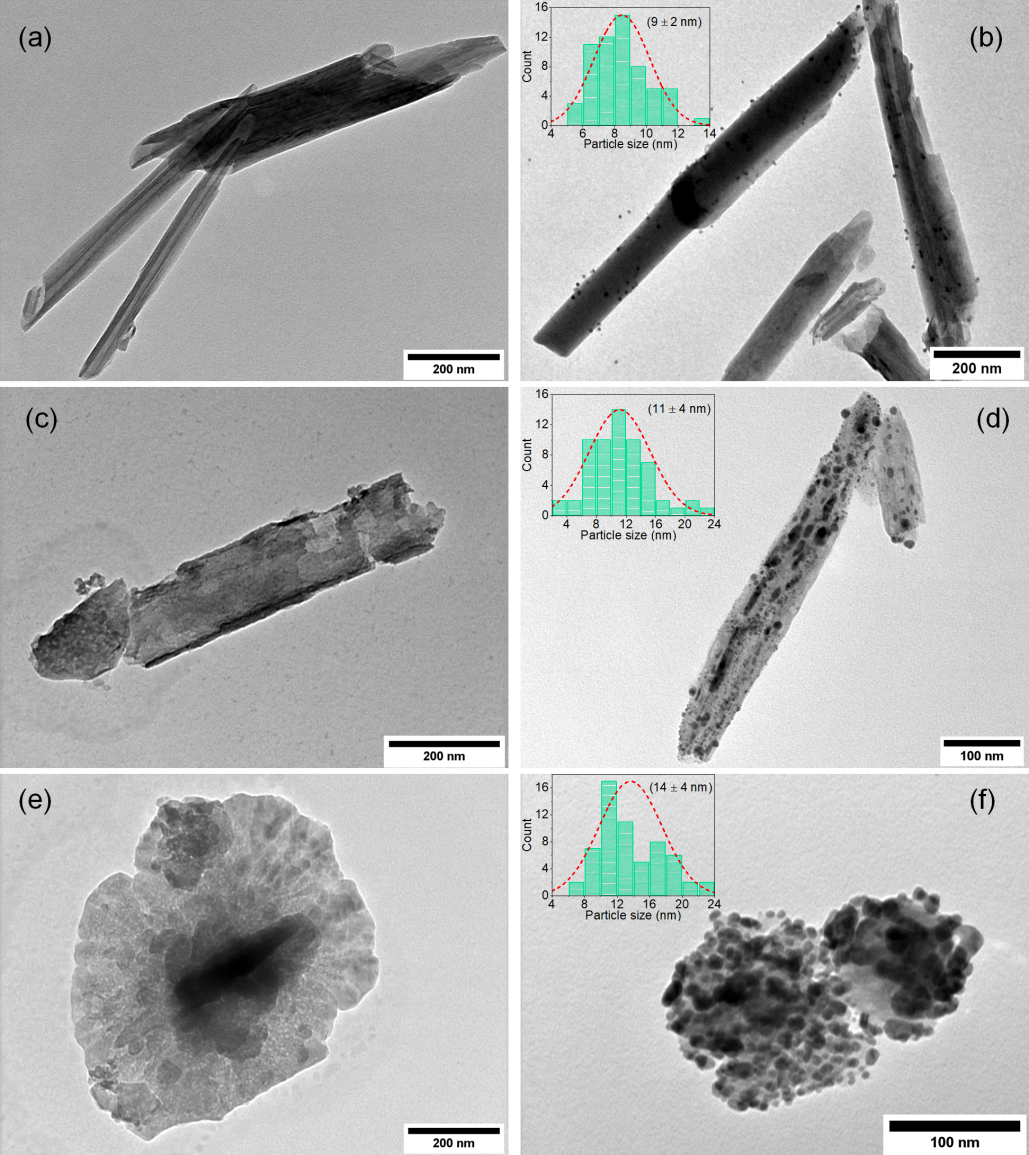
TEM images of (a) HNT-0, (b) Ag/HNT-0, (c) HNT-4, (d) Ag/HNT-4, (e) HNT-8, and (f) Ag/HNT-8. The insets show the corresponding particle size histogram.

Figure 4 shows TEM images of halloysite substrate samples and of samples that went through the thermal reduction of silver. Images of HNT-0, HNT-4, and HNT-8 (Figure 4a,c,e) show that the NaOH treatment induced major structural changes in the HNT substrates. As the treatment progressed, HNT nanotubes slowly unfolded into nanosheets, starting with the enlargement of the central lumen of HNT, as shown in the HNT-4 TEM image (Figure 4c). By day 8, most HNT nanotubes had already turned into nanosheets, as seen in HNT-8 TEM image (Figure 4d). It is known that the initial nanotubular morphology of HNT is a consequence of internal torque in the crystalline structure of the clay [25]. This torque arises from the fact that, while the silicon and aluminol phases of HNT share apical oxygen atoms, the “natural spacing” of the oxygen atoms is different for the two crystalline phases, straining them to conform the oxygen into both structures at the same time. It is our theory that as the NaOH chemical bath etches Al and Si atoms from HNT [31–32], internal sharing of apical oxygen is reduced, diminishing the internal torque to the point where it is no longer strong enough to keep the tubular structure anymore.

TEM images of Ag/HNT-0, Ag/HNT-4, and Ag/HNT-8 (Figure 4b,d,f and the corresponding particle size histograms in the insets) show the distribution of metallic silver nanoparticles nucleated on the HNT surface and also show that nucleation increases with treatment time, resulting in a larger nanoparticles on the surface of Ag/HNT-8 compared to Ag/HNT-4 and Ag/HNT-0, as expected. The particle size distributions show main sizes of 9, 11, and 14 nm for Ag/HNT-0, Ag/HNT-4, and Ag/HNT-8, respectively. The size distribution is more disperse for Ag/HNT-8 compared to Ag/HNT-0 and Ag/HNT-4. Expectedly, the main size difference among the samples is not so large, because the three samples were synthesized under the same conditions, which results in similar nucleation parameters. The small size difference is associated with favouring of the growth stage during particle synthesis, which in turn may be caused by facilitated nucleation and larger surface areas in Ag/HNT-4 and Ag/HNT-8 compared to Ag/HNT-0. EDS results, presented in Table 1, support the TEM analyses by showing that the amount of silver increases with treatment time. These data indicate that the NaOH chemical bath indeed improves the nucleability of silver on the HNT clay surface. Three mechanisms can be suggested to explain the improved silver nucleation in Ag/HNT-4 and Ag/HNT-8, all of them are related to the fact that hydroxy groups are preferential nucleating spots for silver. These mechanisms are: (1) hydroxylation of the HNT surface, that is, by exposing the clay particles to NaOH, hydroxy groups are grafted on the surface [33–35], which will later work as nucleating sites for Ag-NPs; (2) corrosion-induced crystalline defects, that is, by exposing the HNT clay particles to NaOH, a corrosion process is triggered that slowly etches the external crystalline phase [31], inducing defects in the crystalline structure, which expose the internal hydroxy layer of HNT to silver nucleation [23]; (3) unfolding of HNT into nanosheets, that is, as the NaOH treatment proceeds, HNT tubes turn into sheets, which inevitably exposes the inner aluminol layer (rich in hydroxy groups) to silver nucleation (Figure 4).

**Table 1:** EDS results (wt %) for Ag-NPs samples, standard deviation in brackets.

Sample	Ag (wt %)	O (wt %)	Al (wt %)	Si (wt %)

Ag/HNT-0	18.3 (0.4)	49.7 (0.7)	15.6 (0.2)	16.4 (0.2)
Ag/HNT-4	39.8 (2.0)	41.2 (2.1)	9.3 (0.2)	9.7 (0.1)
Ag/HNT-8	46.9 (2.0)	35.2 (1.3)	8.5 (0.1)	9.4 (0.2)

It is not clear which mechanism is the dominant one. Figure 4d and Table 1 indicate that Ag/HNT-4 shows improved nucleability even though its substrate did not unfold. This suggests the first mechanism and the second are already significant enough to induced improved nucleability on the clay surface. While the original intent of the NaOH bath was to graft hydroxy groups onto the HNT surface to improve the formation of Ag-NPs, the opening of the nanotubes into nanosheets was a welcomed surprise, as it exposes the aluminol phase to silver nucleation.

### Thermal reduction of silver nanoparticles

**Figure 5:**
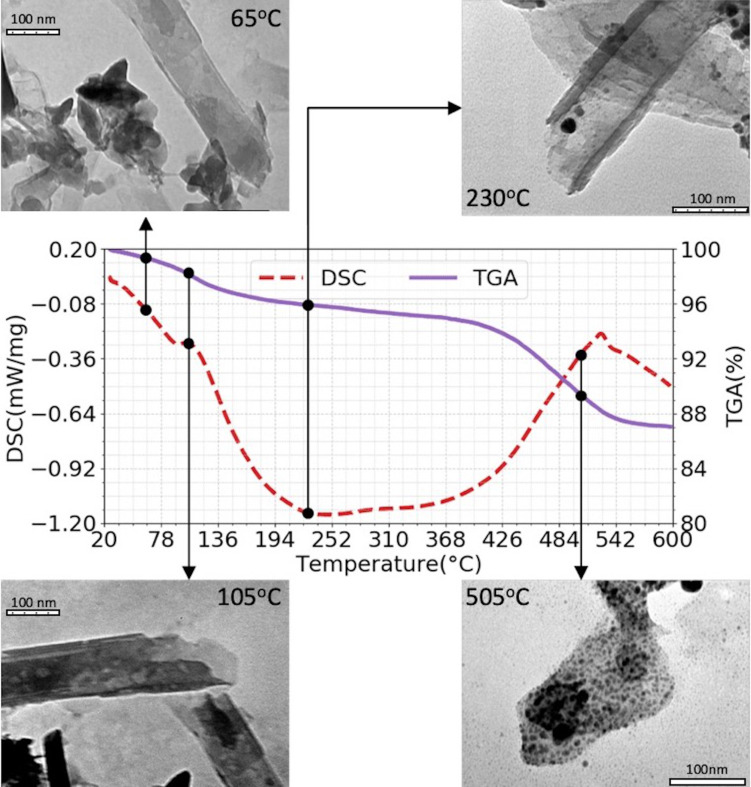
DSC and TGA measurements of Ag-NP formation on HNT-8 substrate from room temperature up to 600 °C in air. TEM image of the samples prepared with the same parameters as those of the DSC/TGA measurements, but the synthesis was stopped at specific temperatures, that is, 65, 105, 230, and 505 °C, to observe the Ag-NP nucleation on the substrates.

**Figure 6:**
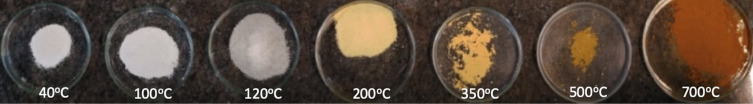
Image of AgNP/HNT-8 with different colours after the synthesis process was interrupted at 40, 100, 120, 200, 350, 500, and 700 °C.

The TGA and DSC analysis results of HNT loaded with silver nitrate are presented in Figure 5. Since the by-products of silver nitrate reduction are oxygen and nitrogen dioxide (both gaseous substances) from the reaction 2AgNO_3_→2Ag_(s)_ + O_2_ + 2NO_2(g)_ [36], we expect to see a mass reduction as AgNP nucleates. Also, because the nucleation is an endothermic process, we expect to see endothermic peaks. Table 2 divides the results into five different thermodynamic phases, characterized by different TGA/DSC behaviour. The TEM images in Figure 5 are meant to correspond to the phases described in Table 2 with structural changes of the material. The colour change during sample heating is presented in Figure 6 and also correlated to the data in Table 2.

**Table 2:** TGA and DSC behaviour as function of the temperature.

Phase	Range (°C)	TGA behaviour	DSC behaviour	Color

I	26–100 °C	slow rate of mass loss	no event	white
II	100–135 °C	mass loss at increased rate	endothermal peak	changing to gray
III	135–420 °C	reduced rate of mass loss	no event	changing to yellow
IV	420–540 °C	mass loss at increased rate	endothermal peak	brown
V	from 540 °C onwards	mass stabilizes (no more losses)	no peak or plateau	orange brown

During phase I (27–100 °C) there is a slight mass loss, which can be attributed to moisture loss and does not correspond to any endothermal or exothermal peak. The corresponding TEM image (at 65 °C in the inset of Figure 5) shows no formation of Ag-NPs. During phase II (100–135 °C) the mass loss increases substantially. The behaviour is concurrent with an endothermal peak. The endothermal behaviour and the fact that the increased rate of mass loss happens near 100 °C (water boiling temperature) strongly suggests that phase II is an evaporation process of humidity left after phase I. Again, the corresponding TEM image (at 105 °C) shows no Ag-NP nucleation. During phase III (135–420 °C) there are no endothermal or exothermal peaks, but there is a low continuous mass loss, associated with a slight change in the color of the compound, suggesting the beginning of Ag-NP formation. Indeed, the corresponding TEM image (at 230 °C) shows some Ag-NPs. According to literature, however, silver nitrate is not expected to thermally decompose below 500 °C [36]. An early formation of Ag-NPs suggests that the modified HNT substrate may have some catalytic effect on the decomposition of AgNO_3_. The low rate of mass loss (about 12–15% at 600 °C) is consistent with a low rate of Ag-NP formation [37]. During phase IV (420–540 °C) there is an increased rate of mass loss and an endothermal peak, now associated with a distinct color change from yellow to brown. This happens near the decomposition temperature of AgNO_3_ [36] and suggests an accelerated reduction of AgNO_3_ into metallic silver (compared to phase III). The corresponding TEM image (at 505 °C) indeed confirms a significant presence of Ag-NPs, indicating that most of the nanoparticle formation occurs during phase IV. This hypothesis is further strengthened by the fact that during phase V (540–600 °C) there is no mass loss and no endothermic peak, suggesting that all AgNO_3_ was already converted into Ag-NPs. Yet, as shown by Figure 6, samples heated above 600 °C still exhibit a colour change. This effect may be due particle growth, which is expected to happen when nanoparticles are heated to higher temperatures.

### Antimicrobial tests

The minimal inhibitory concentration test of AgNP/HNT-8 indicated a MIC of 25 ppm for *E. coli*, and 50 ppm for *S. aureus*, while the MIC of AgNP/HNT-0 was 100 ppm for *E. coli* and 200 ppm for *S. aureus*. The difference in antimicrobial effect against *E. coli* and *S. aureus* can be explained by morphological differences between the two bacteria. As Gram-positive bacteria, *S. aureus* has a thicker cell wall than *E. coli*, and thus the silver ions penetrate into the cells at a lower rate. Those results are consistent with the expected antimicrobial activity of commercial Ag-NPs, that is, a MIC near 10 ppm for Gram-positive/negative bacteria [38]. Results indicate that AgNP/HNT-8 has a stronger antimicrobial effect than AgNP/HNT-0, which can be explained by the higher silver content of AgNP/HNT-8.

**Figure 7:**
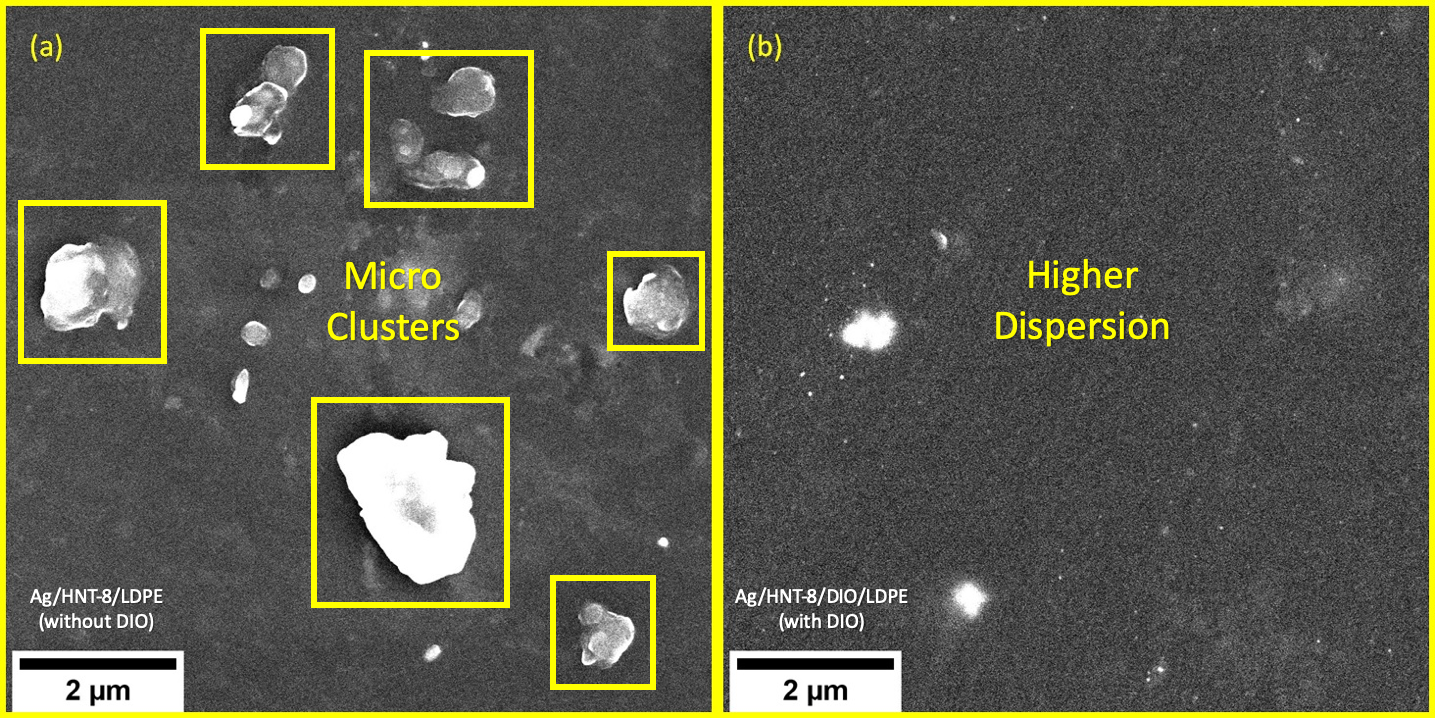
Scanning electron microscopy of (a) Ag/HNT-8-doped LDPE and (b) Ag/HNT-8/DIO-doped LDPE.

The JIS Z 2801 antimicrobial surface activity test shows that 19.64% of *E. coli* colonies and 96.02% of *S. aureus* colonies were able to proliferate into LDPE doped with Ag/HNT-8. The LDPE doped with Ag/HNT-8/DIO showed no signs of proliferation at all, indicating a dramatic increase in antimicrobial activity. Figure 7 shows SEM images of both plastic samples and sheds light on why Ag/HNT-8/DIO exhibited better antimicrobial properties in combination with LDPE. The uncoated sample (Figure 7a) displays the formation of microscopic Ag-NPs aggregates. In contrast, the Ag/HNT-8/DIO-doped sample (Figure 7b) shows a relatively smooth surface, displaying fewer particle clusters and far more nanoscaled dots, suggesting a better dispersion of particles in the LDPE matrix. The antimicrobial properties of Ag-NPs are associated with the release of Ag^+^ ions [39] and this release is correlated with the surface energy of nanoparticles. Thus, the higher antimicrobial surface activity obtained with Ag/HNT-8/DIO is consistent with expectations, as well dispersed nanoparticles tend to have higher surface energy.

**Figure 8:**
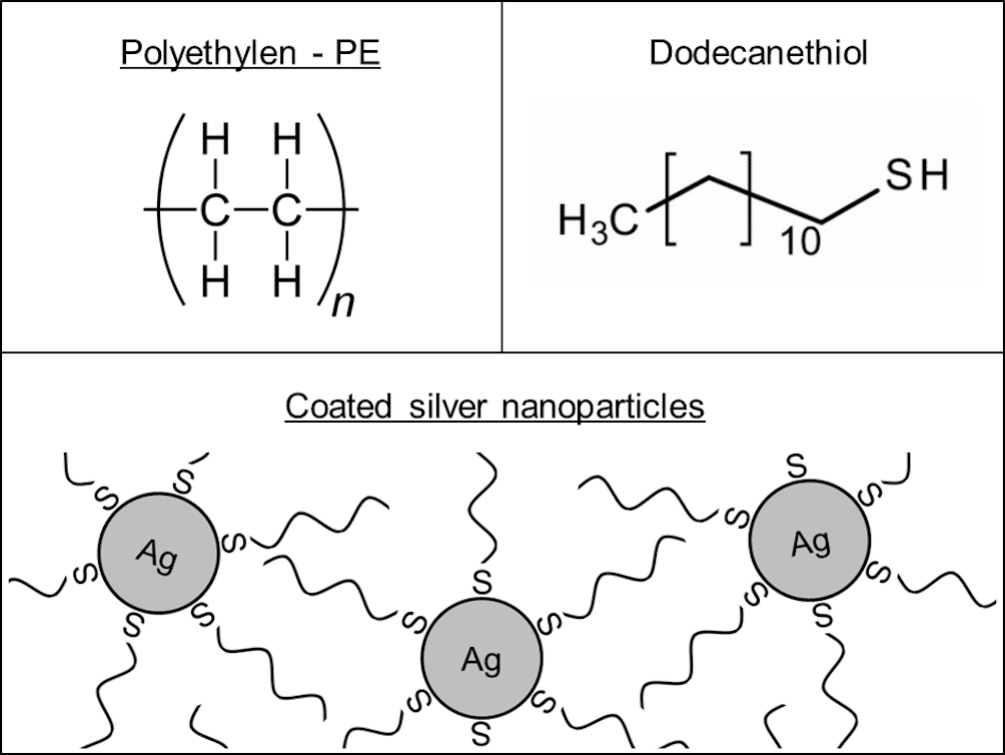
Molecular structures of polyethylene and dodecanethiol, and scheme of Ag-NPs coated with dodecanethiol.

The difference in aggregation between both samples may be explained in terms of matrix polarity. Polymer molecules are polarized to some degree, depending on the type of material. Polyethylene (both low- and high-density) is almost apolar and thus mixes very well with hydrophobic particles and very badly with hydrophilic ones. Qualitative tests have shown that, while Ag/HNT-8 gets wet in water, Ag/HNT-8/DIO is highly hydrophobic. This hydrophobic behaviour is most certainly due to the presence of DIO, a very hydrophobic substance itself. DIO is an organic molecule made of a long carbon chain “tail” attached to a thiol “head” (R-SH), which forms covalent bonds with silver via the sulfur atom. So, by mixing DIO and Ag/HNT-8 one is able to create a thin layer of carbon chains coming out from the Ag/HNT-8 surface (as shown in Figure 8). This yields hydrophobic properties, which result in better dispersability into the LDPE matrix and, consequently, in better antimicrobial surface activity of plastics doped with the DIO-coated sample.

## Conclusion

Treating halloysite with NaOH is an efficient way to enhance silver nucleation on the clay surface. Treated substrates were able to anchor nanosized silver particles, which displayed strong antimicrobial effects against *E. coli* and *S. aureus*. The antimicrobial effect of the treated substrates was higher than that of untreated halloysite. TGA and DSC analysis indicated that most of the reduction of metallic silver from AgNO_3_ occurs at temperatures above 420 °C, while TEM images showed that nucleation at this temperature does not result in uncontrolled grain growth on a microscopic scale. The Ag-NPs produced in this work were also tested for practical applications by dispersing them into LDPE matrices and measuring the surface antimicrobial activity of the resulting composites. While the antimicrobial effect of LDPE doped with “natural” Ag-NPs was disappointing, it was possible to greatly improve the antimicrobial properties by coating the nanoparticles with dodecanethiol, a non-polar surfactant highly compatible with LDPE. This shows that the Ag-NPs synthetized is this work are viable as antimicrobial additives for plastics, probably the most important material for large-scale industrial applications, while also highlighting the importance of controlling chemical affinity between surface and matrix of the nanoparticle, a fact often overlooked in nanoparticle research.

The synthesis process presented in this work uses only common reactants, thermal reduction, and the inexpensive halloysite clay as substrate. It is thus a low-cost solution for antimicrobial nanoparticle production that is also scalable to industrial production.
